# Simultaneous Determination of Multiple Sesquiterpenes in *Curcuma wenyujin* Herbal Medicines and Related Products with One Single Reference Standard

**DOI:** 10.3390/molecules18022110

**Published:** 2013-02-06

**Authors:** Jing-Jing Zhu, Yue-Wei An, Guang Hu, Guo-Ping Yin, Qi-Wei Zhang, Zhi-Min Wang

**Affiliations:** 1 Institute of Chinese Materia Medica, China Academy of Chinese Medical Sciences, Beijing 100700, China; E-Mails: zhujj15@163.com (J.-J.Z.); ayw1987@126.com (Y.-W.A.); huguang@yahoo.com.cn (G.H.); ygp19870526@126.com (G.-P.Y.); zhangqw1955@163.com (Q.-W.Z.); 2 National Engineering Laboratory for Quality Control Technology of Chinese Herbal Medicines, Beijing 100700, China

**Keywords:** *Curcuma wenyujin*, sesquiterpenes, relative response factors, high performance liquid chromatography, quantitative analysis

## Abstract

Some *Curcuma* species are widely used as herbal medicines. Sesquiterpenes are their important bioactive compounds and their quantitative analysis is generally accomplished by gas chromatography (GC) or high performance liquid chromatography (HPLC), but the instability and high cost of some sesquiterpene reference standards have limited their application. It is necessary to find a practicable means to control the quality of herbal medicines. Using one stable component contained in *Curcuma* species to determine multiple analogues should be a practical option. In this study, a simple HPLC method for determination of sesquiterpenes using relative response factors (RRFs) has been developed. The easily available and stable active component curdione was selected as the reference compound for calculating the RRFs of the other eight sesquiterpenes, including zedoarondiol (Zedo), isozedoarondiol (Isoz), aerugidiol (Aeru), (4*S*,5*S*)-(+)-germacrone-4,5-epoxide (Epox), curcumenone (Curc), neocurdione (Neoc), germacrone (Germ) and furanodiene (Fura). Their RRFs against curdione were between 0.131–1.301, with a good reproducibility. By using the RRFs, the quantification of sesquiterpenes in *Curcuma wenyujin* herbal medicines and related products was carried out. The method is especially useful for the determination of (4*S*,5*S*)-(+)-germacrone-4,5-epoxide, curcumenone, germacrone and furanodiene, which often are regarded as the principle components in *Curcuma* species, but unstable when were purified. It is an ideal means to analyze the components for which reference standards are not readily available.

## 1. Introduction

Sesquiterpenes are the major bioactive constituents of *Curcuma* plants, with reported anti-tumor, anti-inflammation, anti-oxidant, arthralgia relieving, anti-thrombosis, and hepatoprotective activities [1,2,3]. More than 130 sesquiterpenes found in *Curcuma* plants have been reported, including twelve structural types such as germacrane, elemane and guaiane, *etc.* [4]. Most pure sesquiterpene compounds are instable and hard to store. Rearrangements and transformations (Cope rearrangements, acid induced cyclizations and photochemical treatments) occur widely in Nature [5]. Under acidic, water and thermal treatments, (4*S*,5*S*)-germacrone-4,5-epoxide cyclized through transannular (T-A) reactions [6]. Furanodiene was found to degrade to curzerene on exposure to heat through a [3.3]-sigmatropic reaction (Cope rearrangement) [7,8]. High purity curcumenone was also unstable when it was isolated from the extract of *Curcuma* plants. Analysis of sesquiterpenes in *Curcuma* plants is generally accomplished by GC or gas chromatography-mass spectrometry (GC-MS) [9], but many heat-sensitive sesquiterpenes cannot be studied by GC-MS as rearrangements take place in the injector, making the components indiscernible by the detector. However, furanodiene, furanoelemene and (4*S*,5*S*)-germacrone-4,5-epoxide were found discernible in *Curcuma* species by HPLC using mild conditions (low temperature, short analysis time) [7,10]. Due to their instability, sesquiterpene reference standards of *Curcuma* plants are expensive and in short supply, which has limited the application of analytical methods for the *Curcuma* plants. For these purposes, it is necessary to determine the relative response factors (RRFs) for the sesquiterpenes using one stable component which is easy to obtain. By establishing the RRFs, it should be a practical way to determine multiple analogues and evaluate the quality of *Curcuma* plants, their extracts and related products. 

RRFs have been adopted in the Chinese, United States and European Pharmacopoeias [11,12,13]. RRFs was successfully used to analyze the chemical composition of tea [14] and herbal medicines containing coumarins [15], anthraquinones [16], steroidal saponins [17], phenanthrenequinones [18] and flavonoids [19], but all of these study objects are stable compounds and there is no report on the application of the RRFs in analysis of sesquiterpenes and their unstable analogues.

*C. wenyujin*, a member of *Curcuma* (Zingiberaceae), is a traditional medicine in China, its raw rhizomes, steamed rhizomes and steamed roots are used as three herbal medicines in the Chinese Pharmacopeia, namely Pian-jiang-huang (PJH), Wen-e-zhu (WEZ) and Wen-yu-jin (WYJ). The oils of these three herbal medicines have anti-tumour [1], anti-inflammatory [2], and antiviral effects in clinic [3]. Suppository of Fufang Ezhuyou (FFEZY) is a preparation which has been used clinically for treatment of annexitis. In this paper, we present a quantitative method for simultaneously determining multiple sesquiterpenes in herbal medicines and related products from *C. wenyujin* by using only one standard reference.

## 2. Results and Discussion

### 2.1. Optimization of Sample Pre-Treatment

Several experiments were carried out in order to optimize sample preparation. The peak areas of sesquiterpenes were investigated under each set of test conditions. Methanol, ethanol, and methanol-water were used for extractions. As a result, 10 mL methanol was chosen as the extract solvent for 0.5 g of sample powder. Extraction methods such as ultrasonic extraction, reflux, and Soxhlet’s extraction were compared. The proper time and extraction temperature were also optimized. The optimal extraction method was obtained by extracting in methanol for 30 min with ultrasonic bath assistance. 

### 2.2. Validation of the Method

**Figure 1:**
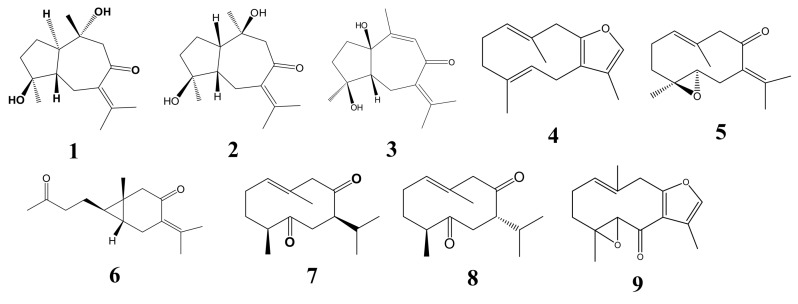
The structures of compounds **1**–**9**.

Calibration graphs for nine sesquiterpenes were constructed using six levels of concentration which covered the concentration ranges expected in the various samples (Figure 1). The characteristics of the calibration curves, including the range of linearity, the square of correlation coefficient (r^2^) of each investigated compound was measured at 244 nm and are listed in Table 1. Excellent linearity was observed with the square of correlation coefficients for all the analyses falling into the range 0.9996–1.0000. The limits of detection (LOD) and quantification (LOQ) were measured based on a signal-to-noise ratio (S/N) at about 3 and 10, respectively. Table 1 showed LOD and LOQ data at 244 nm for each investigated compound.

Validation was assessed by calculating the relative standard deviations (RSDs) and the studies proved that this method had good precision and accuracy (Table 2). Precision was measured by intra-day and inter-day variability. For intra-day tests, a certain concentration solution was analyzed for six times within one day, while for inter-day tests, the solution was examined twice per day for three consecutive days. The RSDs of intra-day and inter-day variability in peak areas of the nine target constituents were less than 1.8% and 2.0%, respectively. The sample solution for all the analytes in 18 hours was stable. To confirm the repeatability, six replicates of sample were extracted and analyzed. The RSDs were less than 1.0%. Recovery test was used to evaluate the accuracy of this method. Three different concentrations (high, medium, low) of the nine standards were added to a certain amount (0.25 g) of the sample with known contents of target analytes. Three replicates were performed for each level test. The samples were prepared following the same procedure and analyzed as described in section 3.2. The recoveries were in the range of 96.2%–100.9% with good RSD (1.5%–3.3%), which indicated that the proposed method had an adequate degree of accuracy for the simultaneous determination of nine target constituents in the samples.

### 2.3. Relative Response Factors

**Figure 2:**
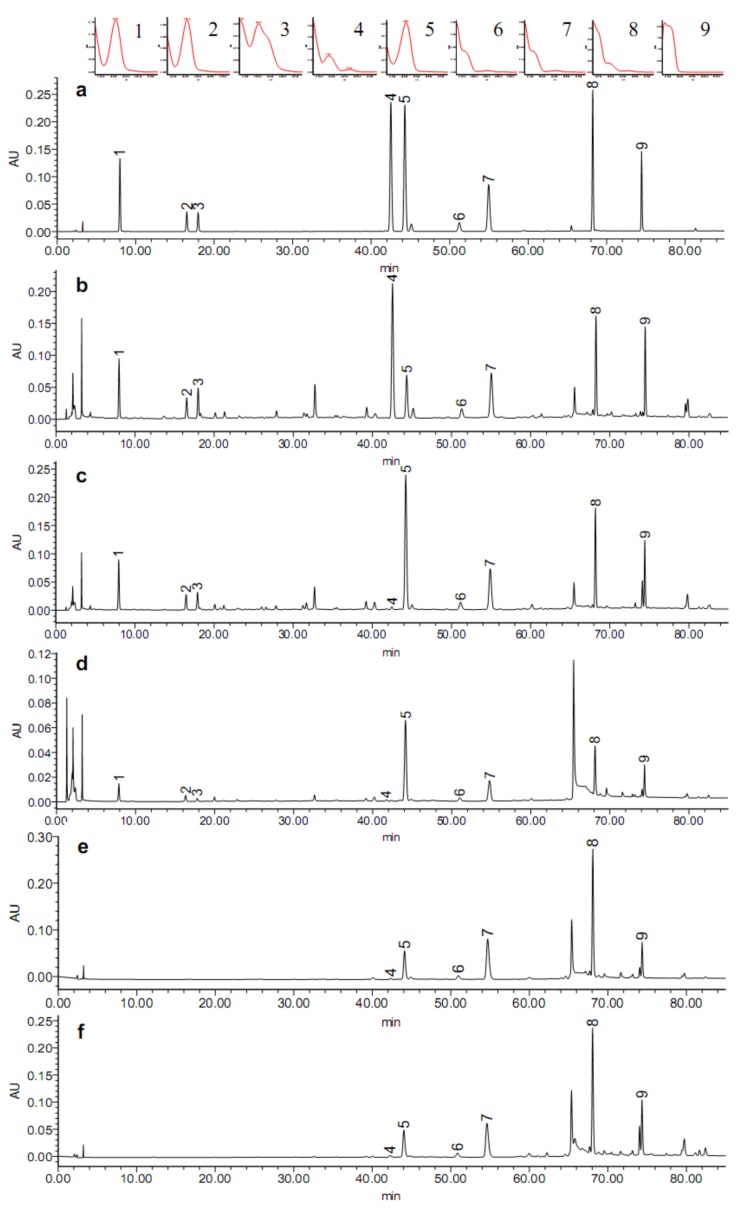
Representative HPLC profiles of the samples of *C. wenyujin* and mixed sesquiterpene standards. (**a**) Mixed sesquiterpene reference standards and their UV spectra; (**b**) the sample of PJH; (**c**) the sample of WEZ; (**d**) the sample of WYJ; (**e**) the essential oil of *C. wenyujin*, and (**f**) commercial sample of suppositories (Fufang Ezhuyou) at 244 nm. (1-zedoarondiol, 2-isozedoarondiol, 3-aerugidiol, 4-(4*S*,5*S*)-germacrone-4,5-epoxide, 5-curcumenone, 6-neocurdione, 7-curdione, 8-germacrone, 9-furanodiene).

Curdione and zedoarondiol were selected as potential reference standards for their stability and availability. The UV absorptions of two compounds are shown in Figure 2. The maximum absorption wavelength of zedoarondiol, isozedoarondiol, aerugidiol, (4*S*,5*S*)-germacrone-4,5-epoxide and curcumenone was about 256 nm, while the maximum absorption wavelength of neocurdione, curdione, germacrone, furanodiene was about 214 nm (Figure 2a). It was found that 244 nm was a good compromise wavelength for detecting all the compounds. The detection wavelength is an important factor influencing the stability of RRFs. Therefore, the relative response factors for the tested sesquiterpenes against curdione and zedoarondiol at 256 nm, 244 nm and 214 nm were listed in Table 3 and Table 4, respectively. Variations in RRFs against curdione and zedoarondiol at 244 nm were less than 5% for all tested compounds, indicating that 244 nm could allow detection of all analytes. The RRFs against curdione at 244 nm were between 0.131–1.301 and the RRFs against zedoarondiol at 244 nm were between 0.528–5.345.

### 2.4. Ruggedness and Robustness Tests of RRF

Different reversed-phase C_18_ columns, namely Diamonsil, Kromasil, Symmetry and two instruments, namely Waters e2695–2998 and Agilent 1260, were used to investigate the variation of the RRFs (Table 5). The results showed that variation of the RRFs at 244 nm was less than that of 256 nm and 214 nm, which further proved that 244 nm was the optimal wavelength. Variation in RRFs against curdione was less than that of zedoarondiol at 244 nm, indicating that curdione was a suitable reference compound. The RRFs had good reproducibility (RSD = 2.3%–4.5%) for different columns and instruments, demonstrating that the RRFs could be used for HPLC quantitative analysis. The stability of the standard solution of curdione was investigated. The standard solution was kept at 4 °C in a refrigerator for 10 days, and the response factors decreased by 1.5% and 1.4%, respectively, indicating that curdione was stable.

### 2.5. Quantitative Measurement of Different Samples from C. wenyujin

In this study, a total of 21 samples of *C. wenyujin* herbal medicines and their related products were collected for HPLC analysis, which included five non-steamed rhizome (PJH), five steamed rhizomes (WEZ), five steamed roots (WYJ), three essential oils and three preparations. Both multiple reference standards and single reference standard methods were used for simultaneously determining nine components in all the samples. These two group results were compared in Table 6. The comparative results of two methods were measured by the value of relative error (RE). The RE of the contents of all the components in tested samples were lower than 10%, which showed that no significant difference was found between two methods, indicating that the RRFs had potential application to evaluate the quality of herbal medicines, extracts and related products. 

And as a result of discrimination for three kinds of herbal medicines derived from *C. wenyujin*, PJH contained a much higher quantity of (4*S*,5*S*)-(+)-germacrone-4,5-epoxide, while WEZ contained more curcumenone, which could used as markers for their quality control. WYJ contained lower contents of all the tested components than those in WEZ and PJH. The proposed method also could be used to evaluate the quality of their oils and preparations. 

## 3. Experimental 

### 3.1. Materials and Reagents

A total of five herbal samples of non-steamed rhizome, steamed rhizome and steamed root of *C. wenyujin*, namely PJH, WEZ and WYJ, were collected from Ruian, Zhejiang province, China in February 2011. All samples were authenticated by authors. The voucher specimen (*Curcuma wenyujin*, No PJH-20110404) had been deposited in the Herbarium of the Institute of Chinese Materia Medica, China Academy of Chinese Medical Sciences, Beijing, China. Besides, the essential oils of *C. wenyujin* were obtained by steam distillation, using a standard apparatus according to the procedures described in Chinese Pharmacopoeia. Then the oils were dried with anhydrous sodium sulphate and stored at 4 °C. In addition, three commercial suppositories of Fufang Ezhuyou (FFEZY) were collected from different pharmaceutical companies in Beijing, China.

HPLC-grade acetonitrile was provided by Thermo Fisher Scientific (Waltham, MA, USA). Water was purified with a Milli-Q system (Millipore, Bedford, MA, USA) and subsequently filtered through a 0.45 µm membrane (Millipore). The other solvents, purchased from Beijing Chemical Factory (Beijing, China), were of analytical grade. Nine reference standards, including zedoarondiol, isozedoarondiol, aerugidiol, (4*S*,5*S*)-(+)-germacrone-4,5-epoxide, curcumenone, neocurdione were isolated by our lab and curdione, germacrone as well as furanodiene were purchased from the National Institute for Food and Drug Control (Beijing, China). The purity for each standard compound was greater than 98% by HPLC analysis. 

### 3.2. Instruments and Chromatographic Conditions

Analysis was performed on two HPLC systems with a Waters 2695–2998 series, including a quaternary pump, a photodiode array detector, a vacuum degasser, a thermostated autosampler, a column compartment, Empower work station, and an Agilent 1260 series, including a quaternary pump, a diode array detector, a vacuum degasser, a thermostated autosampler, a column compartment, a data system (Agilent ChemStation). The chromatographic separation was performed on two Diamonsil C_18_ columns (250 × 4.6 mm, 5 μm), a Kromasil C_18_ column (250 mm × 4.6 mm, 5 µm) and a Waters Symmetry C_18_ column (250 mm × 4.6 mm, 5 µm). The mobile phase consisted of water (A) and acetonitrile (B). The gradient program was as follows: 20% (B) in 0–10 min, 20%–46% (B) in 10–35 min, 46%–48% (B) in 35–50 min, 48%–54% (B) in 50–55 min, 54%–90% (B) in 55–70 min, 90%–100% (B) in 70–75 min and 100% (B) in 75–85 min. The flow rate was kept at 1 mL·min^−1^. Column temperature was kept constant at 35 °C, and the injection volume was 10 µL. The detection wavelengths were 214 nm, 244 nm and 256 nm. Figure 2 showed the HPLC chromatograms of mixed sesquiterpene standards [Figure 2(a)] and the related samples of *C. wenyujin* (Figure 2b–f).

### 3.3. Sample Preparation

Samples of PJH, WEZ and WYJ were powdered to a homogeneous size in a mill, passed through a 40-mesh sieve and dried at 40 °C until constant weight was achieved. Approximately 0.50 g of the pulverized sample was weighed accurately and macerated in 10 mL of methanol. The sample was extracted for 30 min in an ultrasonic bath at 20 °C and the loss of weight due to evaporation of solvent was replenished with methanol. The extract was filtered through a 0.45 µm filter membrane. Then 10 µL of the filtrate was injected into the HPLC system for each analysis.

The essential oils of PJH, WEZ and WYJ (100 mg) were accurately weighed and dissolved in 25 mL methanol (4 mg·mL^−1^). Then it was filtered through a 0.45 µm filter membrane prior to HPLC analysis. The injection volume was 10 µL. 

Commercial suppositories of Fufang Ezhuyou were weighed 0.5 g and dissolved in 10 mL of methanol. Then the next extract procedures were as the same as that of *C. wenyujin* herbal samples mentioned above.

### 3.4. Sesquiterpene Standards Solution Preparation

A mixed stock solution containing reference standards of nine sesquiterpenes was prepared by dissolving weighed accurately each compound in methanol at a concentration of 0.04 mg·mL^−1^ to 1.48 mg·mL^−1^ and diluted to appropriate concentrations for the establishment of calibration curves and relative response factors. Curdione stock solution was prepared by dissolving accurately weighed the compound in methanol at a concentration of 0.15 mg·mL^−1^. Zedoarondiol stock solution was prepared by dissolving accurately weighed the compound in methanol at a concentration of 0.02 mg·mL^−1^.

### 3.5. Calculation of Relative Response Factors and Quantification of Sesquiterpenes

According to the principle of Lambert-Beer, within a concentration range, the absorption of analyte is linearly proportional to sample concentration and their relations can be shown with the formula *C = fA* [18] where *C* is the sample concentration, *A* is the response value, and *f* is the response factor (RF). The value of RF is a constant within a certain linear range. Supposing that several components coexist in a Chinese herbal medicine material sample, every component can be shown as Equation (1). If component *s* is used as a reference substance, the relative response factor (*f_sm_*) between components *s* and *m* is established through Equation (2). Then the third quantitative Equation (3) can be deduced, where *A_s_* and *C_s_* are the peak area and concentration of the reference substance, while *A_m_* and *C_m_* are the peak area and concentration of the target component, respectively. According to Equation (3), if the content of component *s* was authentically determined, the content of component *m* can be calculated through its relative response factor:

(1) $$\frac{C_{i}}{A_{i}}=f_{i}\;(i=1,\;2,\;\ldots ,\;k,\;\ldots ,\;m)$$

(2) $$f_{sm}=\frac{f_{s}}{f_{m}}=\frac{C_{s}.A_{m}}{C_{m}A_{s}}$$

(3) $$C_{m}=\frac{C_{s}A_{m}}{f_{sm}A_{s}}$$

## 4. Conclusions 

The reported assay method uses relative response factors (RRFs) for the determination of nine sesquiterpenes in *Curcuma* species. It is ideal for rapid, routine analysis, especially for those laboratories where sesquiterpene standards are not readily available. With this method, good repeatability of results was obtained, and nine different sesquiterpenes could be determined simultaneously only using one standard reference (curdione). Furthermore, this method is simple, sensitive, robust and accurate, and is applicable to evaluate the quality of three *C. wenyujin* herbal medicines and their related products. It also can be used to quantify the other *Curcuma* species which were reported to contain multiple sesquiterpene analogues.

## Figures and Tables

**Table 1:** Regression data of nine sesquiterpenes in *Curcuma wenyujin* at 244 nm.

Analyte	Calibration equation	Linear range (µg)	r^2^	LOD (ng)	LOQ (ng)
Zedo	y = 9.56 × 10^6^x + 1.59 × 10^3^	0.0236–1.18	0.9999	0.94	2.40
Isoz	y = 9.77 × 10^6^x + 4.36 × 10^2^	0.0081–0.81	1.0000	0.81	2.71
Aeru	y = 1.88 × 10^7^x – 2.15 × 10^3^	0.0040–0.40	0.9999	0.40	1.32
Epox	y = 1.05 × 10^7^x + 9.74 × 10^3^	0.0066–6.58	1.0000	2.60	6.60
Curc	y = 1.61 × 10^7^x + 9.52 × 10^3^	0.0445–4.45	1.0000	0.74	2.23
Neoc	y = 2.31 × 10^6^x – 2.43 × 10^3^	0.0586–2.93	0.9999	1.20	2.90
Curd	y = 2.41 × 10^6^x + 3.06 × 10^3^	0.296–14.79	1.0000	2.40	5.91
Germ	y = 8.04 × 10^6^x + 2.34 × 10^3^	0.059–2.98	0.9999	0.39	0.98
Fura	y = 1.65 × 10^6^x + 1.07 × 10^4^	0.137–6.85	0.9996	0.90	2.34

**Table 2:** Precision, stability, repeatability and recovery of investigated components.

Analytes	Precision		Stability	Repeatability		Accuracy	
	Intra-day RSD/%	Inter-day RSD/%	18 h RSD/%	Concentration (mg∙g^−1^)	RSD/%	Recovery/%	RSD/%
Zedo	0.70	1.8	0.66	1.061	0.68	100.9	3.3
Isoz	0.51	1.7	0.81	0.396	0.90	98.4	3.7
Aeru	0.61	2.0	0.82	0.292	0.76	96.8	1.8
Epox	0.58	0.59	1.0	4.838	0.81	98.1	1.5
Curc	0.58	0.86	0.66	0.783	1.0	100.6	2.5
Neoc	0.48	1.5	0.48	1.843	0.68	99.2	2.9
Curd	1.8	1.4	1.7	9.875	0.72	97.8	1.8
Germ	1.0	0.70	1.4	2.720	1.0	96.2	2.5
Fura	0.66	1.3	1.3	7.618	1.0	100.9	2.2

**Table 3:** The relative response factors (RRFs) for sesquiterpene standards against curdione.

Analytes	256 nm ^a^		244 nm ^b^		214 nm ^c^	
	RRF	RSD/%	RRF	RSD/%	RRF	RSD/%
Zedo	0.022	4.5	0.249	2.4	3.698	0.89
Isoz	0.024	3.8	0.246	0.48	5.976	8.2
Aeru	0.015	3.0	0.131	1.0	0.612	6.2
Epox	0.039	4.3	0.226	2.0	0.904	0.49
Curc	0.015	4.4	0.147	1.8	2.289	4.5
Neoc	1.089	4.4	1.105	2.8	1.034	3.7
Curd	1.000	0.00	1.000	0.00	1.000	0.00
Germ	0.047	4.7	0.297	2.0	0.249	0.46
Fura	1.697	13.0	1.301	3.4	0.289	3.2

^a^ The maximum absorption wavelength of zedoarondiol, isozedoarondiol, aerugidiol, (4*S*,5*S*)-germacrone-4,5-epoxide and curcumenone; ^b^ The compromise wavelength for all analytes; ^c^ The maximum absorption wavelength of neocurdione, curdione, germacrone, furanodiene.

**Table 4:** The relative response factors (RRFs) for sesquiterpene standards against zedoarondiol.

Analytes	256 nm ^a^			244 nm ^b^			214 nm ^c^	
	RRF	RSD/%		RRF	RSD/%		RRF	RSD/%
Zedo	1.000	0.00		1.000	0.00		1.000	0.00
Isoz	1.085	1.7		0.986	2.0		1.626	8.2
Aeru	0.695	2.7		0.528	1.6		0.165	5.3
Epox	1.769	0.26		0.906	0.45		0.245	1.4
Curc	0.686	0.20		0.591	0.68		0.619	3.6
Neoc	48.935	9.4		4.328	4.2		0.287	5.6
Curd	45.192	4.5		4.017	2.5		0.270	0.89
Germ	2.133	0.49		1.194	0.41		0.067	1.3
Fura	78.789	15.4		5.345	4.7		0.078	2.4

^a^ The maximum absorption wavelength of zedoarondiol, isozedoarondiol, aerugidiol, (4*S*,5*S*)-germacrone-4,5-epoxide and curcumenone; ^b^ The compromise wavelength for all analytes; ^c^ The maximum absorption wavelength of neocurdione, curdione, germacrone, furanodiene.

**Table 5:** Variations of RRFs on different columns and instruments.

	256 nm				244 nm				214 nm			
	Zedo		Curd		Zedo		Curd		Zedo		Curd	
	RRF ^a^	RSD/% ^b^	RRF	RSD/%	RRF	RSD/%	RRF	RSD/%	RRF	RSD/%	RRF	RSD/%
Zedo	1.000	0.00	0.022	10.5	1.000	0.00	0.253	4.2	1.000	0.00	3.697	0.93
Isoz	1.092	1.6	0.024	9.0	0.993	1.9	0.251	3.5	1.665	9.3	6.146	12.1
Aeru	0.696	2.6	0.015	8.3	0.524	2.5	0.132	2.3	0.163	2.9	0.603	2.9
Epox	1.795	1.6	0.040	9.9	0.914	1.9	0.230	3.8	0.246	0.54	0.910	0.74
Curc	0.693	2.3	0.015	9.1	0.604	3.9	0.150	3.0	0.617	3.7	2.239	1.5
Neoc	51.195	22.5	1.138	16.3	4.299	6.0	1.092	3.2	0.277	4.6	1.016	4.6
Curd	44.187	8.5	1.000	0.00	3.969	5.0	1.000	0.00	0.272	0.49	1.000	0.00
Germ	2.161	1.9	0.048	9.0	1.217	3.3	0.305	3.1	0.067	1.8	0.249	1.1
Fura	84.812	10.1	1.679	18.3	5.451	14.3	1.309	4.5	0.077	3.1	0.286	2.4

^a^ The average of relative response factors on different columns and instruments; ^b^ The relative standard deviations of the relative response factors on different columns and instruments.

**Table 6:** Comparison of sesquiterpenes contents in *Curcuma* samples determined by two methods (mg∙g^−1^, n = 2).

Samples	Zedo			Isoz			Aeru			Epox			Curc			Neoc			Curd	Germ			Fura		
	1 ^b^	2 ^c^	RE/% ^d^	1	2	RE/%	1	2	RE/%	1	2	RE/%	1	2	RE/%	1	2	RE/%	2	1	2	RE/%	1	2	RE/%
PJH-1 ^a^	1.02	0.968	5.1	0.405	0.397	2.0	0.281	0.277	1.4	6.07	5.95	2.0	0.874	0.865	1.0	2.07	2.03	1.9	10.82	2.44	2.42	0.82	8.32	8.17	1.8
PJH-2	1.63	1.55	4.9	0.664	0.648	2.4	0.423	0.418	1.2	11.25	11.02	2.0	1.26	1.24	1.6	3.26	3.18	2.5	16.93	5.20	5.14	1.2	15.9	15.60	1.9
PJH-3	0.767	0.729	5.0	0.286	0.281	1.8	0.178	0.177	0.56	3.90	3.83	1.8	0.619	0.614	0.81	1.43	1.41	1.4	6.93	1.64	1.63	0.61	5.56	5.48	1.4
PJH-4	1.35	1.29	4.4	0.527	0.515	2.3	0.456	0.450	1.3	7.92	7.75	2.1	1.22	1.20	1.6	2.90	2.82	2.8	14.33	4.07	4.03	0.98	12.57	12.33	1.9
PJH-5	1.08	1.03	4.6	0.421	0.412	2.1	0.347	0.343	1.2	6.04	5.92	2.0	0.898	0.888	1.1	2.05	2.01	2.0	10.55	3.00	2.98	0.67	8.24	8.10	1.7
WEZ-1	0.754	0.717	4.9	0.284	0.280	1.4	0.181	0.180	0.55	0.119	0.123	3.4	3.44	3.38	1.7	1.65	1.62	1.8	8.78	2.41	2.39	0.83	7.56	7.43	1.7
WEZ-2	0.453	0.432	4.6	0.160	0.159	0.63	0.085	0.086	1.2	0.088	0.093	5.7	2.07	2.04	1.4	1.16	1.15	0.86	5.55	1.36	1.35	0.74	3.64	3.61	0.82
WEZ-3	0.668	0.635	4.9	0.239	0.236	1.3	0.135	0.134	0.74	0.223	0.225	0.90	3.55	3.49	1.7	1.68	1.65	1.8	9.73	2.43	2.41	0.82	6.77	6.66	1.6
WEZ-4	0.826	0.784	5.1	0.313	0.308	1.6	0.160	0.159	0.63	0.226	0.228	0.88	4.41	4.33	1.8	1.84	1.80	2.2	9.98	2.67	2.65	0.75	6.24	6.15	1.4
WEZ-5	0.638	0.606	5.0	0.233	0.230	1.3	0.191	0.190	0.52	1.75	1.72	1.7	1.82	1.79	1.6	1.90	1.86	2.1	9.21	2.80	2.78	0.71	9.20	9.04	1.7
WYJ-1	0.133	0.128	3.8	0.057	0.059	3.5	–	–	–	–	–	–	0.479	0.477	0.42	0.296	0.314	6.1	1.15	0.256	0.261	2.0	0.718	0.753	4.9
WYJ-2	0.150	0.145	3.3	0.068	0.070	2.9	–	–	–	–	–	–	0.216	0.219	1.4	0.472	0.484	2.5	0.559	–	–	–	–	–	–
WYJ-3	0.158	0.152	3.8	0.068	0.070	2.9	–	–	–	–	–	–	0.744	0.737	0.94	0.352	0.368	4.5	1.88	0.580	0.581	0.17	1.59	1.61	1.3
WYJ-4	0.148	0.143	3.4	0.058	0.060	3.5	0.022	0.023	4.5	0.076	0.082	7.9	0.698	0.692	0.86	0.267	0.286	7.1	2.09	0.818	0.816	0.24	2.00	2.01	0.50
WYJ-5	0.265	0.254	4.2	0.105	0.105	0.00	0.051	0.052	2.0	0.093	0.098	5.4	0.393	0.393	0.00	0.277	0.296	6.9	2.02	0.711	0.711	0.00	1.44	1.46	1.4
EO-1	– ^e^	–	–	–	–	–	–	–	–	0.672	0.665	1.0	16.48	16.24	1.5	19.66	18.94	3.7	217.4	93.77	92.72	1.1	100.3	103.9	3.6
EO-2	–	–	–	–	–	–	–	–	–	–	–	–	9.53	9.39	1.5	21.59	20.80	3.7	238.2	104.6	103.4	1.1	115.5	119.6	3.5
EO-3	–	–	–	–	–	–	–	–	–	0.323	0.319	1.2	5.41	5.33	1.5	12.90	12.42	3.7	245.32	134.7	133.2	1.1	123.4	127.7	3.5
FFEZY-1	–	–	–	–	–	–	–	–	–	0.087	0.087	0.00	1.10	1.08	1.8	1.51	1.45	4.0	12.75	6.45	6.38	1.1	10.91	11.29	3.5
FFEZY-2	–	–	–	–	–	–	–	–	–	0.074	0.074	0.00	1.09	1.07	1.8	1.53	1.48	3.3	12.57	5.72	5.66	1.0	7.02	7.26	3.4
FFEZY-3	–	–	–	–	–	–	–	–	–	0.470	0.465	1.1	0.976	0.962	1.4	2.51	2.41	4.0	19.99	7.93	7.85	1.0	7.04	7.29	3.6

^a^ PJH-1 to PJH-5 represented the samples of non-steam rhizomes, WEZ-1 to WEZ-5 represented the samples of steamed rhizomes, WYJ-1 to WYJ-5 represented the samples of steamed root, EO-1 to EO-3 represented the essential oil derived from *C. wenyujin*, and FFEZY-1 to FFZEY-3 represented the Suppositories of Fufang Ezhuyou (FFEZY); ^b^ The contents were determined by single reference standard method; ^c^ The contents were determined by multiple reference standards method; ^d^ Relative error (%) = (content determined by single reference standard method − content determined by multiple reference standards method)/content determined by single reference standard method ×100. ^e^ Not detected.
